# Advances in objective assessment of ergonomics in endoscopic surgery: a review

**DOI:** 10.3389/fpubh.2023.1281194

**Published:** 2024-01-05

**Authors:** Shuang Liu, Yuan-you Li, Dan Li, Feng-Yi Wang, Ling-Jie Fan, Liang-xue Zhou

**Affiliations:** ^1^Mianyang Central Hospital, School of Medicine, University of Electronic Science and Technology of China, Mianyang, China; ^2^Department of neurosurgery, West China Hospital of Sichuan University, Chengdu, China; ^3^College of Computer Science, Sichuan University, Chengdu, China; ^4^School of Communication and Information Engineering, Chongqing University of Posts and Telecommunications, Chongqing, China; ^5^Department of rehabilitation medicine, West China Hospital of Sichuan University, Chengdu, China; ^6^The Fifth People’s hospital of Ningxia, Ningxia, China

**Keywords:** occupational health, endoscopic surgery, ergonomics, surgeon, review

## Abstract

**Background:**

Minimally invasive surgery, in particular endoscopic surgery, has revolutionized the benefits for patients, but poses greater challenges for surgeons in terms of ergonomics. Integrating ergonomic assessments and interventions into the multi-stage endoscopic procedure contributes to the surgeon’s musculoskeletal health and the patient’s intraoperative safety and postoperative recovery.

**Objective:**

The purpose of this study was to overview the objective assessment techniques, tools and assessment settings involved in endoscopic procedures over the past decade and to identify the potential factors that induce differences in high workloads in endoscopic procedures and ultimately to design a framework for ergonomic assessment in endoscopic surgery.

**Methods:**

Literature searches were systematically conducted in the OVID, pubmed and web of science database before October 2022, and studies evaluating ergonomics during the process of endoscopic procedures or simulated procedures were both recognized.

**Results:**

Our systematic review of 56 studies underscores ergonomic variations in endoscopic surgery. While endoscopic procedures, predominantly laparoscopy, typically incur less physical load than open surgery, extended surgical durations notably elevate ergonomic risks. Surgeon characteristics, such as experience level and gender, significantly influence these risks, with less experienced and female surgeons facing greater challenges. Key assessment tools employed include electromyography for muscle fatigue and motion analysis for postural evaluation.

**Conclusion:**

This review aims to provide a comprehensive analysis and framework of objective ergonomic assessments in endoscopic surgery, and suggesting avenues for future research and intervention strategies. By improving the ergonomic conditions for surgeons, we can enhance their overall health, mitigate the risk of WMSDs, and ultimately improve patient outcomes.

## 1 Introduction

With the advancement of science and technology, the terminology of “minimally invasive” has penetrated into various fields of surgical procedures. Endoscopic surgery, in particular, has the tremendous benefits of a smaller incision, little discomfort, and a quick recovery (1), which optimizes the enhancement of patients’ quality of life and recovery and significantly lessens patients’ suffering. However, a crucial participant in this successful change, the surgeon, has borne considerable unforeseen costs and ergonomic constraints (2). Surveys on laparoscopic surgery, for instance, have revealed that 73 to 100% (3) of surgeons who conduct standard laparoscopic surgery have WMSDs (Work-related Musculoskeletal Disorders), with a range of 73 to 88% for complaints (4). This indicates a growing pandemic of patient benefit and physician misery. Unfortunately, endoscopic surgeons seldom come out to disclose injuries to their providers despite being aware of the higher physical demands and discomfort they encounter during surgery.

The development of WMSDs in endoscopic surgeons is complex and multifaceted. Unlike other occupations, several unique factors contribute to WMSDs in this group. Prolonged static positions commonly assumed during procedures lead to muscle fatigue and an elevated risk of WMSDs (5). The repetitive nature of endoscopic tasks, such as instrument manipulation and precise motor skills, can cause overuse injuries and musculoskeletal strain (6). Beyond physical strain, these procedures demand intense concentration and focus, leading to cognitive fatigue. This mental load can impair attention to proper body mechanics and temporarily mask muscle fatigue, contributing to a hazardous working environment. Furthermore, the interplay between physical and mental fatigue not only affects the surgeons but also poses a potential risk to patient safety. Fatigued surgeons operating in ergonomically challenging conditions are more likely to make errors, thereby compromising the safety of the procedures they perform (7). This connection underscores the urgent need for comprehensive strategies in healthcare that address both the physical and cognitive aspects of surgeon workload to enhance surgeon well-being and ensure patient safety.

Ergonomics is the scientific discipline which applies theory, principles, data and methods to design and optimize human well-being (the overall health and quality of life) and overall system performance (8). In the past, ergonomic assessment and intervention studies have typically focused on industrial manufacturing, however, it has recently been discovered that surgeons tend to work in harsher environments and working conditions than some industrial workers (9).

For endoscopic surgery, ergonomics can be integrated into all stages of the endoscopic surgery unit to provide a safe and comfortable ergonomic environment design for surgeons and patients (10), including preoperative surgeon scheduling, simulation training, and protocol planning; intraoperative layout and optimization of surgical instruments and patient position design; and postoperative safety care and interventions for occupational musculoskeletal disorders for endoscopic surgeons, as well as investment in construction of surgical instruments and surgical technology.

Ergonomics-based assessments assist in the early detection of environmental and individual potential factors on surgeons’ musculoskeletal health (11) and propose specific interventions. While workloads assessment has relied on paper-based subjective questionnaires over the years, with the development of information technology and human factors engineering, some objective assessment methods have emerged and have proven to be more thorough and sensitive to the identification of characterization of WMSDs (12). Motion analysis, force platform, and biochemical parameters such as surface EMG are beginning to be used in endoscopic surgery analysis of objective ergonomics for surgeons; and combined with subjective questionnaire collection to obtain high reliability assessment results.

Accordingly, given the explicit information extracted from the existing literature, the purpose of the work in this review is to analyze objective ergonomic assessment studies in endoscopic surgery to (a) ascertain the sources of heterogeneity and the threat of reproducibility, (b) identify and categorize the potential factors that induce differences in workloads, (c) summarize sound assessment frameworks, assessment instruments, assessment tools, and assessment settings, and (d) suggest future trends.

## 2 Methods

### 2.1 Search strategy and inclusion criteria

In order to achieve full coverage screening of the medical and scientific literature and to ensure that we capture a wide range of relevant research, literature searches were performed in the medical and engineering field, including pubmed, OVID databases of EMBASE, and Web of Science, for studies published from the earliest date until 2022, which ensures the reliability of included data by avoiding premature access to potentially unverified or incomplete research findings for 2023, safeguarding the robustness of our analysis while maintaining temporal consistency in our review process. Our search utilized a including of (1) Ergonomics, (2) Endoscopy, (3) Muscle strain, (4) workload, and (5) Surgeons as search strings. The inclusion and exclusion criteria of this review are listed as follows:

1. Studies had to be published with an accessible full text and written in either English, German, or Spanish.
2. The type of surgery involved in this review must be relevant to the surgical endoscopy and traditional open surgery and robotic-assisted surgery was not included in the review. When several different surgical procedures were performed in a study, at least one of those procedures is required to involve surgical endoscopy. For instance, it should be included when a study attempts to compare the ergonomic risk between endoscopic surgery and robot-assisted surgery.
3. Studies had to conclude statement about objective indicators, evaluation techniques, and conclusions, which were relevant to posture, electromyography and other physiological parameters. Studies that only based arbitrary questionnaires were disallowed.

### 2.2 Data extraction

Two researchers independently conducted the literature search, screening, and data extraction. Any disagreements regarding eligibility or data extraction were settled through discussion with an additional reviewer. A data extraction form was designed to store the data from the original literature, which included the following variables: publication year, sample characteristics (number of surgeons, age, and gender), type of surgery, study design, objective ergonomic risk assessment indicators, assessment sites and tools, Consolidation of subjective investigations and the outcome of the ergonomics.

## 3 Results

### 3.1 Literature search and selection

**Figure 1:**
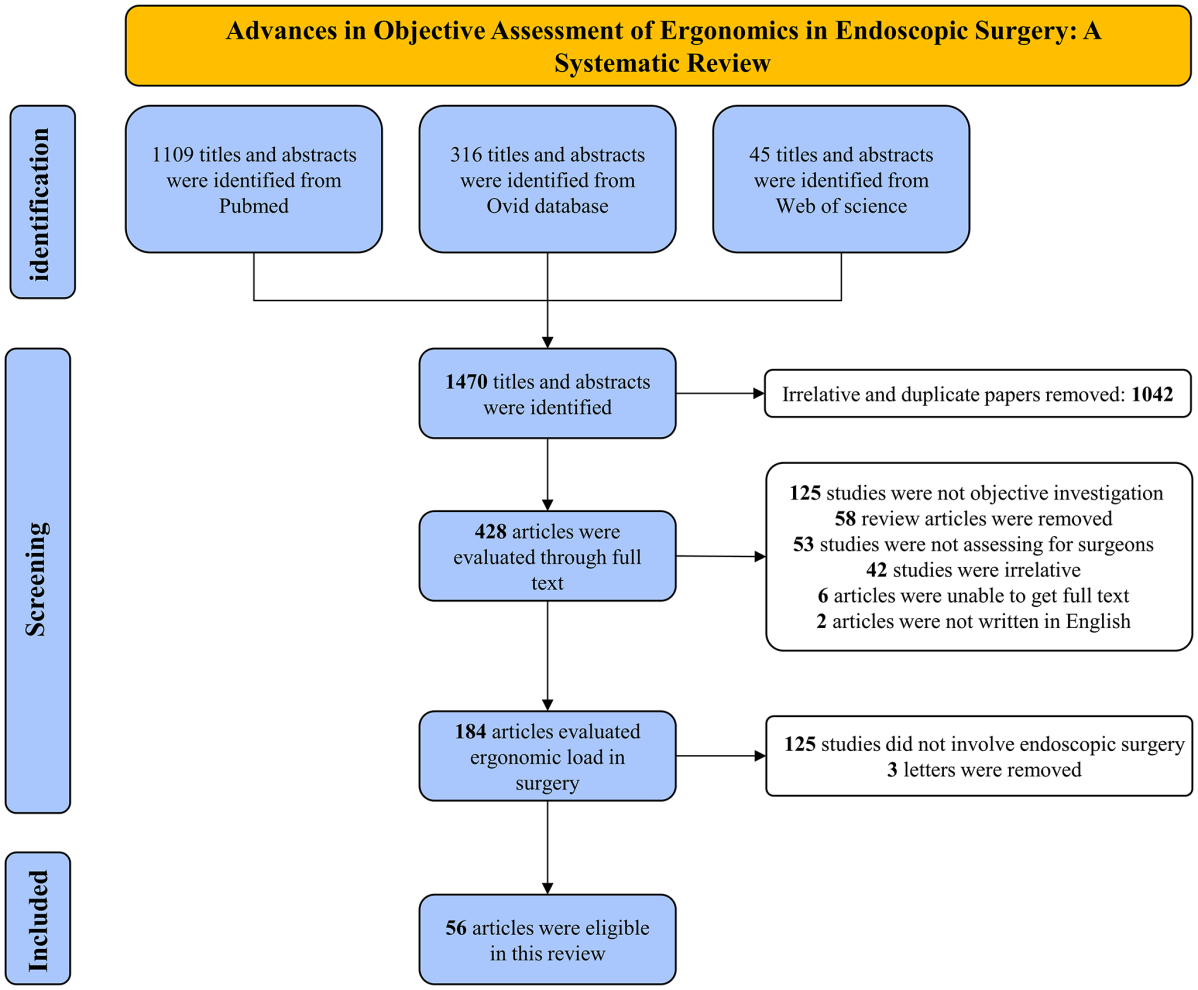
Articles selection flowchart.

From our literature search, we identified 1,470 titles and abstracts. These were then methodically examined against specific inclusion and exclusion criteria, tailored to align with our research questions and objectives. The criteria focused on aspects like study design, target population, measured outcomes, and their pertinence to the surgical practice being studied. This rigorous examination led to the selection of 428 articles, which were then subjected to a detailed full-text analysis to further evaluate their relevance and contributions to our research. The following considerations led to the exclusion of a total of 362 articles: (1) There was no objective assessment employed; (2) It was a review of the literature; (3) The research participants were not surgeons; (4) Endoscopy was not used during the procedure; (5) The paper was not available; and (6) Language. Ultimately, a total of 56 studies were included in this systematic review (Figure 1).

### 3.2 Characteristics of studies

This review included 56 studies in total, 34 (60.7%) studies were from North America and 15 (26.8%) studies were from Europe. The preponderance of the publication years were from 2017 to 2022 (69.6%), with an upward trend. In addition, the number of surgeons participating were recorded with a median of 9.5.

Table 1 offers a summary of the studies that were included. Of all the endoscopic surgical techniques employed in the surgeries, laparoscopy was the most frequent (*n* = 45), followed by, colonoscopy (*n* = 4), nasal endoscopy (*n* = 3), shoulder arthroscopy (*n* = 1), laryngoscopy (*n* = 1), thoracoscopy (*n* = 2), and hysteroscopy (*n* = 1). The types of surgeries primarily included conventional endoscopic surgeries, robot-assisted endoscopic surgeries with endoscopy (*n* = 14). Some open surgeries were incorporated in order to engage in comparisons (*n* = 3).

**Table 1:** Summary characteristics of included studies.

First author	Year	N	Surgery type	Country	Subjective	Experimental places		Evaluation parameters		
						Reality	Simulator	Posture	EMG	Others
Liang (13)	2019	5	Laparoscopic surgery	USA	NASA-TLX	x			x	
Sers (14)	2021	6	Laparoscopic surgery	UK	NI		x	x		
Arrighi-Allisan (15)	2022	6	Functional endoscopic sinus surgery	USA	NASA-TLX	x		x		
Ramakrishnan (16)	2017	2	Functional endoscopic sinus surgery	USA	NASA-TLX/PDQ	x			x	
Lobo (17)	2019	6	Bilateral endoscopic sinus surgery	Spain	NMQ/PDQ	x		x		
Dalager (18)	2020	13	Laparoscopy and robotic-assisted laparoscopic surgery	Denmark	Borg CR-10	x		x	x	
Armijo (19)	2022	18	Laparoscopic surgery	USA	PFS-12	x			x	
Kratzke (20)	2022	10	Laparoscopic surgery	USA	Custom survey		x	x		
Rodrigues (21)	2020	26	Laparoscopic surgery	USA	PFS-12		x		x	
Zihni (22)	2016	1	Laparoscopic surgery	USA	NI	x			x	
Thurston (23)	2022	5	Foregut laparoscopic surgery	USA	NASA-TLX	x			x	
Lowndes (24)	2019	17	Laparoscopic cholecystectome	USA	NASA-TLX/VAS	x				x
Dalager (25)	2019	6	Robotic-assisted laparoscopic surgery	Denmark	NMQ	x		x	x	
Lohre (26)	2020	2	Arthroscopic shoulder surgery	Canada	NI	x		x		
Shiang (27)	2022	27	Upper and lower endoscope	USA	NI	x			x	
Armijo (28)	2019	16	Laparoscopy and robotic-assisted laparoscopic surgery	USA	PFS-12	x			x	
Dalsgaard (29)	2020	12	Laparoscopy and robotic-assisted laparoscopic surgery	Denmark	NMQ/Borg CR-10	x			x	
Zárate (30)	2019	31	Laparoscopy and robotic-assisted laparoscopic surgery	USA	NASA-TLX	x			x	
Szeto (31)	2013	2	Robotic-assisted and laparoscopic rectal surgery	HK	NI	x			x	
Zihni (32)	2014	1	Laparoscopy and robotic-assisted laparoscopic surgery	USA	NI	x			x	
Monfared (33)	2022	20	Laparoscopy and robotic-assisted laparoscopic surgery	USA	BQ/TQ	x		x	x	
Hubert (34)	2013	11	Laparoscopy and robotic-assisted laparoscopic surgery	France	NASA-TLX/Borg CR-10	x			x	x
Shergill (35)	2021	12	Colonoscopies	USA	Borg CR-10	x			x	
Lee (36)	2010	4	Colonoscopies	USA	NI	x		x		
Abdelrahman (37)	2016	1	Laparoscopic cholecystectomy	USA	Surg-TLX	x		x		x
Hallbeck (38)	2017	6	laparoendoscopic single-site surgery	USA	NASA-TLX/Borg CR-10		x	x		
Riggle (39)	2015	24	Laparoscopic surgery	USA	SURG-TLX/Borg CR-10		x	x	x	
Lee (40)	2014	13	Laparoscopy and robotic-assisted laparoscopic surgery	USA	NASA-TLX		x		x	
Yang (41)	2021	24	Laparoscopic and open surgery	USA	NASA-TLX	x		x		
Dai (42)	2021	1	Laparoscopy and robotic-assisted laparoscopic surgery	China	NASA-TLX	x			x	
Wang (43)	2017	1	Laparoscopic and open surgery	USA	NI	x			x	
Pazouki (44)	2017	62	Laparoscopic cholecystectomy procedures	Iran	NMQ	x		x		
Hignett (45)	2017	42	Laparoscopic hysterectomy	UK	Custom survey		x	x		
Athanasiadis (46)	2021	20	Laparoscopic surgery	USA	Custom survey	x		x	x	
Bartnicka (47)	2018	NI	Laparoscopic cholecystectomy	Poland	NI	x		x		
McCrory (48)	2012	24	Laparoscopic and laparoscopic single-site surgery	USA	6-Likert scale		x	x		
Nieboer (49)	2013	26	Laparoscopic surgery	NLD	NI		x		x	
Yu (50)	2016	8	Laparoscopic surgery	USA	NASA-TLX		x	x		
Moss (51)	2020	4	Laparoscopy and robotic-assisted laparoscopic surgery	UK	NI		x	x	x	
Sánchez-Margallo (52)	2014	50	Laparoscopy	Spain	NI		x	x		
Pérez-Duarte (53)	2013	30	Laparoscopic dis-section and suturing maneuvers	Spain	NI		x		x	
Yang (54)	2020	53	Surgeries across different specialties	USA	Borg scale	x		x		
Khan (55)	2020	30	Colonoscopy	Canada	NI		x	x		
Baird (56)	2021	8	Flexible laryngoscopy and awake laryngeal surgeries	USA	Verbally evaluation		x	x	x	
Asadi (57)	2021	12	Laparoscopic surgery	USA	BQ/Likert scale	x			x	
Yurteri Kaplan (58)	2018	27	Vaginal hysterectomy	USA	Borg CR-10	x		x		
Ordóñez-Ríos (59)	2017	7	Laparoscopic cholecystectomy	Cuenca	NMQ	x		x		
Steinhilber (60)	2017	57	Laparoscopic surgery	Germany	NI		x	x	x	
Yoon (61)	2016	1	Thoracoscopic pulmonary lobectomy	Korean	NI	x			x	
Zihni (32)	2014	6	Laparoscopy and robotic-assisted laparoscopic surgery	USA	NI		x		x	
Hardy (62)	2021	3	Laparoscopic and open surgery	Ireland	NI	x		x		
Shergill (63)	2009	3	Colonoscopy	USA	NI	x			x	
Pace-Bedetti (64)	2019	13	Laparoscopic surgery	Spain	NI		x	x		
Butler (65)	2013	6	Laparoscopy and robotic-assisted laparoscopic surgery	USA	VAS	x		x	x	
Lim (66)	2021	3	Thoracoscopic & laparoscopic surgery	Korea	NASA-TLX	x		x	x	
Zhang (67)	2017	14	laparoscopic cholecystectomy	China	NI		x		x	x

*N* represents the number of surgeons; NI representative is not mention.

Although the objective of this systematic review focused on ergonomics quantitative methods for endoscopic surgeries, up to 62.5% (*n* = 35) of the studies used a conjunction analysis of subjective questionnaires to evaluate the ultimate results simultaneously. Eleven types of questionnaires were applied in these studies, consisting mainly of investigative questionnaires for musculoskeletal disorders/discomfort, and immediate questionnaires to evaluate workload or fatigue.

NASA Task Load Index (NASA-TLX) was the most utilized questionnaire in these studies (*n* = 13) which allows users to perform subjective workload in mental demand, physical demand, temporal demand, performance, effort and frustration (68).

Objective assessment of ergonomics was based on electromyographic (*n* = 34) and motion analysis (*n* = 30). Four studies used other tools such as heart rate and salivary cortisol as ergonomics indicators and 12 studies combined more than 2 instruments for the assessment of ergonomics study designs tended to be cross-sectional (*n* = 23) and cohort (*n* = 27), while RCT were utilized in 5 research and case–control was only employed in 1 interventional study.

### 3.3 Methodology of objective assessment in endoscopic ergonomics

#### 3.3.1 Potential factors of ergonomic risk difference

Potential factors leading to a higher ergonomic risk were grouped into three categories: (1) Work task related factors; (2) Characteristics (surgeon and patient)-related factors; and (3) Environment-related factors.

##### 3.3.1.1 Work task

The work task related load consists mainly of different types of surgeries, surgical equipment, assignment of surgical tasks, and duration and number of surgeries.

###### 3.3.1.1.1 Surgery type

In the included literature, 20 studies focused on ergonomics differences between surgeons or surgeon teams due to different types of procedures, especially focus on the differences among open, endoscopic and robotic-assisted surgery. Among comparative studies of traditional open versus laparoscopic surgery, one study measured the activation of the upper body muscles during the laparoscopic and open phases of sigmoid colectomies and demonstrated that the laparoscopic surgery provides ergonomic benefits in several upper muscle groups compared to the open surgery (41). The average neck and trunk angle was shown to be considerably higher for open surgery than for laparoscopic surgery in two studies (41, 54) And when compared to laparoscopic surgery, open surgery involved much longer time spent in the physically taxing torso position (54). In contrast to these views, a study explored the sagittal and rotational movements of the neck in laparoscopic surgery versus open surgery. The results of the study showed that laparoscopic surgery required significantly less skin stretching during flexion and rotational movements compared to open surgery, which demonstrated that laparoscopic surgery requires a longer period of static neck position (62). Robot-assisted surgery can improve the flexibility of surgical operations, reduce tremors, and optimize the surgeon’s workload and physical strain based on ergonomic principles. Several studies have indicated superior performance in terms of postural stability (65), cumulative muscle strain (40), workload (34), and physical demands (29) for robot-assisted surgery compared to conventional endoscopic surgery. Notably, while robot-assisted surgery reduces postoperative discomfort and muscle strain in the upper extremity for the surgeon, it increases static neck positioning and back stiffness compared to endoscopy. Additionally, forearm muscles, particularly those that manage the ulnar offset movements of the wrist joint, exhibit a considerable level of muscle exertion during robot-assisted surgery (31).

###### 3.3.1.1.2 Surgical equipment

A possible reason for the musculoskeletal symptoms reported by surgeons is the ergonomic limitations of the surgical handles designed for surgeon (69). All surgeons involved in such procedures are at risk for complications in the left and right wrist, right thumb, and left thumb. The left thumb and both wrists exceed the limits of movement and are at risk for repetitive motion injuries (63). Five studies explored handle settings in endoscopic surgery and examinations were included in this systematic review, and they focused on laparoscopic and colonoscopic handles. Some endoscopes that are more biomechanically compatible have been developed and have been shown to be beneficial in reducing the ergonomics on the surgeon (24, 39, 50). It is worth noting that the angular configuration of the endoscope (50), the height difference between the endoscope and the table, and the area of the operating field (60) all contribute to the occurrence of ergonomic risk differences. Veelen et al. have identified the ideal relationship between surgeon height and patient abdominal wall (PAW) location (70). The anterior claw’s tip need to be positioned between 70 and 80% of the surgeon’s elbow height. The suggested table height is deduced from the fact that the abdominal sagittal depth is contained at intervals of 30 to 40 cm. Furthermore, a study confirmed that laparoscopic tools with adjustable handle angles decrease the ergonomic risk of musculoskeletal strain and allow alternating tasks between floor and ceiling positions without compromising surgical performance (50).

###### 3.3.1.1.3 Duration of surgery

Colonoscopy procedures, thoracoscopic lobectomies, and a comparative study of open and laparoscopic surgeries all highlight the relationship between surgical duration and ergonomics. In colonoscopies, which average about 19.5 min, significant muscle loads in the forearms and excessive pinch forces can pose ergonomic risks, suggesting that even shorter surgeries can be demanding due to repetitive tasks (63). Thoracoscopic lobectomies, lasting around 99 min, show notable muscle fatigue, especially in muscles like the lumbar erector spinae due to prolonged static postures, highlighting the cumulative effect of longer surgeries (61). Furthermore, a comparative study of open and laparoscopic surgeries reveals that longer procedures in both types lead to increased self-rated fatigue and pain, particularly in the neck and lower back. Open surgeries pose a higher postural risk due to larger neck and torso angles (41). This underscores the need for ergonomic interventions in surgeries, as surgeons often maintain high-risk postures for extended periods, affecting various body parts irrespective of the surgery type or duration.

##### 3.3.1.2 Characteristics (surgeon and patient)

A comprehensive review of fourteen studies revealed several key findings regarding the impact of surgeons’ characteristics on their physical and psychological loads. These studies identified gender disparities, with results showing that women surgeons experience more physical load and fatigue during surgeries compared to their male counterparts (19). Variations in surgical experience also influence ergonomic loads, as surgeons with less experience or fewer years in their career were found to face higher physical strain (15, 23, 44). In addition, surgeon assistants and operating room nurses are subjected to severe ergonomic loads comparable to those experienced by surgeons (22, 44). The studies also examined the effect of surgical posture, revealing slight differences in ergonomic load between standing and sitting positions during endoscopic procedures. Specifically, in functional nasal endoscopy, surgeons face different workloads risks depending on whether they are standing or sitting, with sitting posing a greater risk to the upper extremity and standing to the legs (15, 16).

Furthermore, five studies investigated the relationship between patient characteristics and ergonomic load on surgeons. These studies focused on factors such as patient Body Mass Index (BMI) and positioning during surgery. It was found that performing laparoscopic surgery on patients with high BMI resulted in increased non-neutral postures and musculoskeletal discomfort for surgeons (14, 51). Contrarily, some studies reported no significant differences in ergonomic stress or workload when operating on obese versus non-obese patients (13). Additionally, the patient’s positioning during surgery was found to affect surgeon ergonomics, as demonstrated in a study where the lateral position during shoulder arthroscopy posed a higher ergonomic risk to orthopedic surgeons compared to the beach chair position (26).

##### 3.3.1.3 Environment-related factors

Environment-related factors within the operating room (OR) significantly influence the ergonomic experience of endoscopic surgeons. The positioning of assistant surgeons, the arrangement of video displays, and the layout of surgical components and pedals are pivotal. Moreover, the height and design of operating beds and chairs, along with the ergonomics of surgical tools and handles, play a crucial role in surgeon performance and comfort (59). Head equipment, like headlamps and magnifiers, though essential, can contribute to musculoskeletal strain and ergonomic stress, necessitating careful design considerations to reduce the physical burden on surgeons during endoscopic procedures (3, 71).

#### 3.3.2 Ergonomics assessment tools

##### 3.3.2.1 Motion analysis-based

The optimum surgical posture aids in the optimization of the surgeon’s physical load and the evaluation and generalization of these postures lie under the purview of motion analysis. To objectively quantify postural load, 30 studies have tried to capture, track, and analyze surgeons’ actions intraoperatively, based on a perspective of motion analysis. Generally, intraoperative posture data acquisition relies on optical sensors and inertial sensors, the former including conventional cameras, depth structured light cameras, and optical tracking systems. Using a standard camera to record a surgeon’s surgery or taking interval photos is one of the most archaic techniques. One representative of a structured light camera sensor is the Kinect. The most recent version of Azure Kinect is outfitted with an orientation sensor for sophisticated computer vision model development, a 12 megapixel full HD camera, and a 1 megapixel improved depth camera. It has body segmentation capabilities that can produce an anatomically accurate skeleton that contains either the entire or the parts of the body.

A study used an optical tracking system to compare the kinematic differences between surgeons with straight and curved instruments (38). Optical tracking systems typically require participants to wear passive mirror-reflective markers or active LEDs and operate with the aid of high-speed cameras to reconstruct the virtual human body as a real-time action.

By integrating dispersed IMUs (Inertial Measurement Units) into the wearable accessory, the inertial sensor—which is made up of an angle meter, an accelerometer, and a gravimeter—allows for the capturing of human motion. IMUs from APDM Wearable Technologies was most frequently employed among the included researches (*n* = 5), and Perception Neuron from Noitom was also be employed (*n* = 1).

For angular motion capture of particular locations, the inertial measurement unit can also be utilized by itself. In order to detect motion tracking and body angles in the surgeon’s upper body, Monfared and Athanasiadia positioned the IMU sensors on the surgeon’s torso, head, chest, low back, and right and left biceps (33, 46) (Table 2).

**Table 2:** Posture and motion tracking-based ergonomic risk assessment.

First author	Standard	Posture capture and analysis tool
Sers (14)	LUBA	IMUs sensor (Noitom) & Kinect
Arrighi-Allisan (15)	Ideal joint angles (from REBA)	IMUs sensor (APDM Wearable Technologies)
Lobo (17)	RULA	Kinect
Dalager (18)	RULA	Manual observation and recording
Kratzke (20)	RULA	Video recording and analysis
Dalager (25)	RULA	Manual observation and recording
Lohre (26)	Joint angles	Video recording and analysis
Monfared (33)	Ideal joint angles (from RULA)	IMUs sensor (APDM Wearable Technologies)
Lee (36)	Joint angles	Biaxial electrogoniometers
Hallbeck (38)	Joint angles	Raptor-12 Motion Camera
Riggle (39)	Joint angle (wrist deviation)	Goniometer (Biometrics SX230 and SG65)
Yang (41)	Ideal joint angles (from RULA)	IMUs sensor (APDM Wearable Technologies)
Pazouki (44)	RULA	Video recording and analysis
Hignett (45)	REBA	AO
Athanasiadis (46)	Ideal joint angles (from RULA)	IMUs sensor (APDM Wearable Technologies)
Bartnicka (47)	Joint angle (wrist deviation)	Wireless electrogoniometers (TEA CAPTIV T-Sens)
McCrory (48)	Joint angles	Biaxial electrogoniometers (Wrist and elbow angular)
Yu (50)	Joint angles	Raptor-12 Motion Camera
Moss (51)	Amount of the movement	IMUs sensors(6 Waseda Bioinstrumentation system)
Sánchez-Margallo (52)	Hand and wrist positions & RULA	Data glove (CyberGlove)
Yang (54)	Joint angles (from RULA)	IMUs sensor (APDM Wearable Technologies)
Khan (55)	RULA&REBA	Video recording and analysis
Baird (56)	RULA	Photo documentation
Yurteri-Kaplan (58)	Joint angles	Video recording
Ordóñez-Ríos (59)	REBA	Video recording
Steinhilber (60)	Wrist joint angles	Twin axis goniometer
Hardy (62)	Neck flexion, extension and rotation	Wireless strain gauge monitor (SELS Bodyguard 105TM)
Pace-Bedetti (64)	Joint angles and displacements	Video recording
Butler (65)	BESS	Nondominant, single-leg stance
Lim (66)	REBA	Video recording

AO represents artificial recording and calculation.

Ergonomic assessment tools such as electrogoniometers are also widely used when researchers are more concerned with localized high loads on the human body (*n* = 4). Furthermore, when exploring the effects of laparoscopic handles on surgeon physical loads, researchers typically focus more on surgeon forearm, elbow, wrist, and finger activities. Hence, it is also desirable to capture the surgeon’s hands motion utilizing data gloves (*n* = 1), which are consists of a series of conductive sensors with sensitive resistive flow that detects changes in bending and is capable of recording the relative angle of deflection of the hand.

Commonly analyzed indicators include joint angles and ergonomics rating scales. Ergonomics scales incorporate joint angles of body subcomponents (e.g., trunk, neck, legs, upper arms, lower arms and wrist) and are evaluated in conjunction with the parameters of force, coupling, and duration. Of the included literature, RULA (Rapid upper larms assessment) is the most frequently used ergonomics rating scale (*n* = 12), followed by REBA (*n* = 5) and LUBA (*n* = 1). RULA, as the most widely used ergonomic scale, is created exclusively for light labor and concentrates on the classification of postural risk for the upper limb (72). REBA is a postural analysis system that is sensitive to musculoskeletal concerns in a variety of occupations, particularly in the health care and other service industries (73). Unlike RULA, REBA divides the joint movements of the entire body into several groups. One study measured ergonomics based on the LUBA scale, which was selected for the presence of prolonged low-risk postures and intermittent high-risk postures in laparoscopic surgery, which is consistent with the properties of LUBA for medium-risk settings (74). In general, higher ergonomics scores often indicate greater risk exposure and alertness to the need for urgent change.

When the motion data is being analyzed, ergonomists or occupational therapists can use intraoperative video/image data captured by the camera to manually generate ergonomics scores. Additionally, certain recent computer vision-based techniques can be utilized to extract human skeleton postures from videos and automatically determine ergonomic scores. A study was conducted to evaluate the body positioning of residents during laparoscopic surgery, and a computer vision algorithm based on Openpose and SMPL-X was designed for estimating the RULA score from 2D images/videos (20). To acquire the necessary joint angle data, the raw data captured by the inertial sensors typically needs to be filtered, smoothed, and denoised. These processes are carried out directly within the Matlab program, and some advanced IMUs solutions additionally offer software platforms for automated analysis. It is significant to mention that before any official measurements can be taken, all sensors must be calibrated beforehand in the environment.

##### 3.3.2.2 sEMG-based

The endoscopic surgeon’s awkward posture, keeping the arm in an elevated position, and the use of high level strength instruments all cause an accumulation of muscle stresses that can cause microtrauma to the muscle tissues, leading to inflammation, pain, and ultimately, tissue damage. If the accumulated tissue damage exceeds the body’s ability to repair itself, it can lead to chronic musculoskeletal disease. Therefore, understanding muscle fatigue induced by surgical procedures in endoscopic surgeons can help guide the development of targeted interventions.

One of the important tools to measure muscle fatigue is electromyography (EMG), EMG signals are frequently used to assess muscle fatigue and ergonomics in occupational settings. Traditionally, invasive needle electrodes were used to obtain EMG signals, but recent advancements have made wireless surface EMG sensors the preferred method due to their convenience and accuracy.

EMG signals can be analyzed in both the time and frequency domains. Following signal acquisition, rectification, smoothing, and maximum voluntary contraction (MVC) normalization are typically performed. Commonly used metrics in the time domain include the integrated EMG value (iEMG) and root mean square (RMS). iEMG represents the intensity of muscle activity over a certain period, while RMS describes the average variation in surface EMG amplitude. In the frequency domain, median frequency (mDF) and mean power frequency (MPF) are commonly used metrics. MVC% is also a widely used EMG parameter that represents the percentage of actual EMG amplitude to the EMG amplitude at maximum voluntary contraction. Other metrics such as amplitude probability distribution function (APDF) and relative activation time (RAT) are also used.

Among the muscle groups involved in the included literature (Table 3), the most frequent EMG assessment regarding the trapezius muscle (*n* = 24) was followed only by the deltoid muscle (*n* = 19). In addition, the biceps, triceps erector spinae, radial wrist flexors, finger extensors, superficial finger flexors, cervical erector spinae, and wrist extensors were also frequently present. Interestingly, most of the included literature focused on Upper Extremity muscles, with only 1 study on thoracoscopic lobectomy and mediastinal lymph node dissection for lung cancer that included the rectus femoris and anterior tibialis muscles of the lower limb (61). A study quantifying and comparing surgeons’ ergonomic pressures during laparoscopic versus open access for sigmoid resection found that degree of surgical difficulty may have less impact laparoscopically compared to open access (43).Furthermore, a study to assess differences in physical load among surgeons with different surgical experience collected EMG activity of surgeons’ upper extremity muscles during laparoscopy, demonstrating higher RVC and MF levels intraoperatively for less experienced surgeons (23).

The muscle activation of the upper trapezius (UT), anterior deltoid (AD), and radial carpal flexor (FCR) muscles during the surgeon’s execution of the robot-assisted surgery phase showed a higher level of activation compared to performing a conventional laparoscopic procedure (28). In addition, one study found female had greater muscle effort than male in laparoscopic surgery (19).

##### 3.3.2.3 Other tools-based

In addition to the feature of surface EMG signal as a biomarker of ergonomics, several other non-invasive biomarkers have been used to quantify the ergonomics of endoscopic surgeons. Previous studies have indicated that heart rate variability markers in the frequency domain, such as low frequency and high frequency, can give some indication of changes in physical and psychological loads. Moreover, mean heart rate and mean heart rate “cost” (intraoperative heart rate – resting heart rate) were also employed in a study to investigate the difference in workload between standard LAP and Robot-assisted surgery, and ultimately found that standard laparoscopic surgeons had increased heart rates compared to robot-assisted surgery (34). Salivary cortisol levels, a stress hormone, were employed in two trials to measure the ergonomics on laparoscopic surgeons. At various points during the process, they collected surgeon saliva using collection aid. The samples were then frozen, and at the conclusion of the trial, they were thawed and examined (24, 37), and both studies ultimately found that surgeons’ salivary cortisol levels were significantly higher during single-incision laparoscopic cholecystectomy than during conventional laparoscopic surgery. Eye tracking data, especially pupillary dilation, are typically sensitive to task difficulty and workload (75), and Zhang measured the psychological load on participants during the procedure by recording the physiological parameters of their eyes through an oculomotor while performing the task in the laparoscopic simulator (67).

#### 3.3.3 Evaluation places

The integrity and validity of ergonomic assessment outcomes hold profound significance in surgical practice, demanding meticulous consideration of the chosen assessment site. Notably, 64.3% of the studies conducted meticulous trials within real operating room (OR) settings, while 35.7% (*n* = 20) employed surgical simulators to explore ergonomic intricacies. The authenticity of real-life OR surgery exposes surgeons to genuine occupational challenges, thereby rendering assessment outcomes more robust and clinically applicable.

Nonetheless, real-life OR surgery is not devoid of challenges that necessitate attention. Intrusion of ergonomic data capture instruments may inadvertently divert the surgeon’s focus; wearable instruments could contribute to an additional physical burden; the stringent requirement of sterilization for OR instruments might jeopardize the integrity of sophisticated equipment; and patient reluctance poses another practical hurdle. To circumvent these intricacies, the implementation of cadaveric surgery emerges as an attractive alternative. Intriguingly, nasal endoscopy studies based on cadaveric surgery, instead of clinical surgery, have discerned ergonomic disparities related to surgeon posture, ultimately yielding reliable conclusions (16, 17). Cadaveric surgery, although akin to real-life OR conditions, offers an exceptionally high-fidelity replication of clinical surgery’s tactile and visual aspects. It’s noteworthy that cost considerations have motivated certain researchers to compare robot-assisted laparoscopic surgery to traditional laparoscopic procedures using animal models (34, 42).

In parallel, surgical simulators emerge as instrumental tools, distinguished by their economic viability, controlled environmental factors, and ready accessibility. Within the realm of laparoscopy-related investigations, open-up simulators, box simulators, and virtual reality simulators are recurrently employed, primarily for training and preoperative assessment purposes. Open-up simulators cater to fundamental tasks encompassing handle movement, object manipulation, suturing, and cutting. Progressively complex, box simulators replicate realistic surgical settings, encompassing cannula needles, light sources, and real-time visual feedback. Virtual reality simulators, harnessed by panoramic computer vision and force feedback, simulate abdominal images with remarkable realism akin to genuine OR scenarios, affording opportunities for intricate surgical simulations.

Delving into the clinical realm, distinctions in upper extremity fatigue are perceptible between laparoscopic and robotic surgical training environments (40). In the study’s scope, a laparoscopic box simulator was juxtaposed against a robotic surgical training simulator, emulating the da Vinci system. Furthermore, investigations addressing surgeon postural load and muscle strain within box simulators employing foam of varying thicknesses to mimic patient BMI underscore the clinical relevance of ergonomic research (14, 51). Beyond laparoscopy, specialized simulators for nasal endoscopy and colonoscopy have been developed, enhancing training and assessment precision (55, 56).

In evaluating the ecological validity of simulations used in ergonomic assessments, it is crucial to recognize both their strengths and limitations in mimicking real surgical environments. Simulations offer a controlled, replicable setting for detailed ergonomic analysis, allowing researchers to isolate and study specific variables in depth. This controlled environment is particularly beneficial for initial training and for studying the ergonomic impact of specific tasks or equipment under standardized conditions. However, the ecological validity of these simulations is inherently limited by their inability to fully replicate the dynamic and often unpredictable nature of actual surgical procedures. Real-life surgeries involve variables such as patient-specific anatomical challenges, intraoperative complications, and varying team dynamics, which are difficult to reproduce in a simulated setting (76). Moreover, the stress and pressures experienced by surgeons in actual operations, which can significantly impact ergonomic load and cognitive function, are often attenuated in simulated environments.

#### 3.3.4 Proposed framework for endoscopic ergonomics research

**Figure 2:**
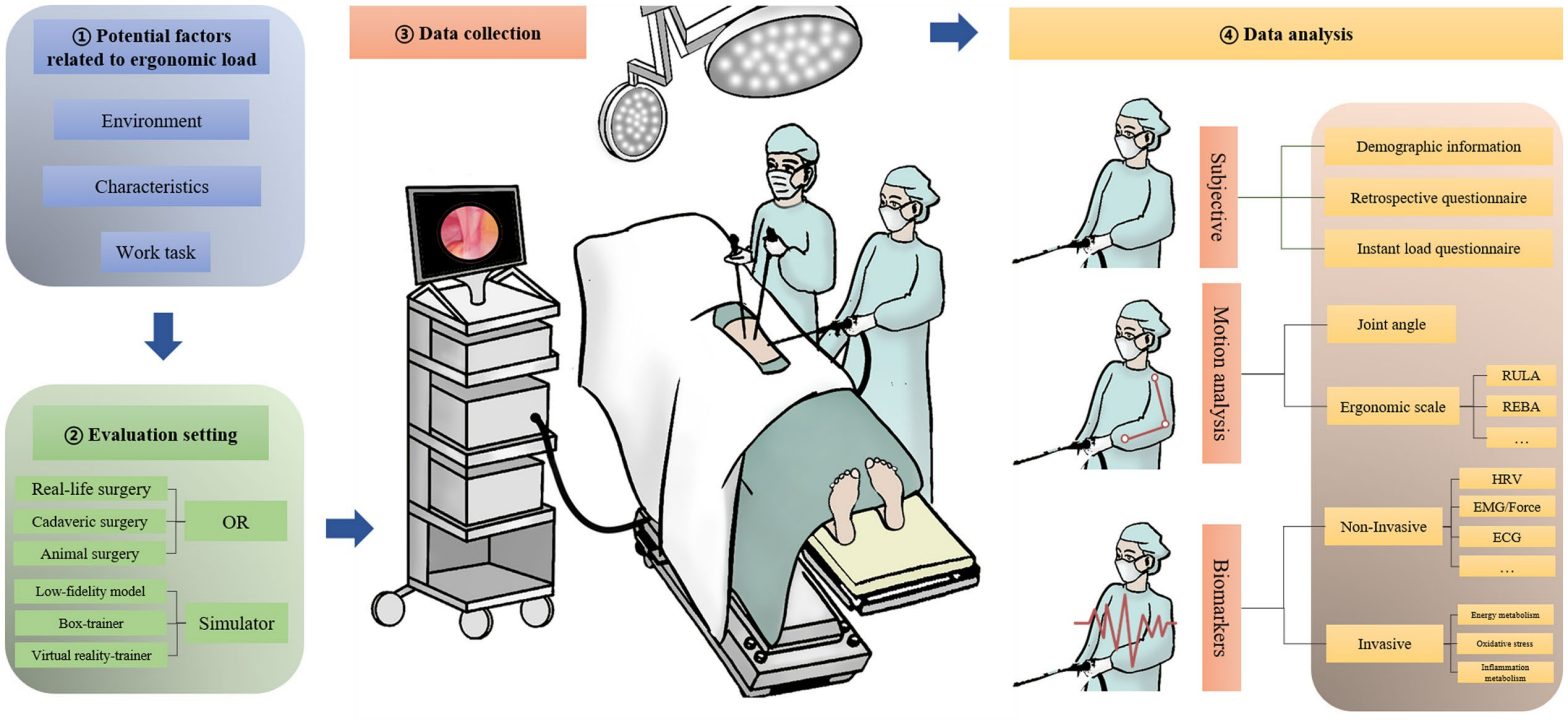
Structure of ergonomics assessment in endoscopic surgery.

The comprehensive framework developed in this study, as depicted in Figure 2, is grounded in the critical evaluation of ergonomic assessment methodologies analyzed in previous sections. It systematically organizes the findings into a cohesive structure aimed at enhancing ergonomic evaluations. The “Identification of Factors” component draws from the diverse variables discussed earlier, such as surgeon’s posture and patient BMI, and situates them within a broader context affecting workload. “Selection of Evaluation Setting” takes into consideration the detailed examination of various settings, from ORs to simulations, acknowledging their respective strengths and limitations. The “Data Collection Methodology” integrates the diverse data types, from subjective questionnaires to objective measures like EMG, discussed throughout the review. This meticulously crafted framework thus serves as a testament to the synthesis of our findings, presenting a pathway for deploying these insights into practical, real-world applications that address the ergonomic challenges identified.

### 3.4 Interventions to reduce workload

Ergonomic assessment forms the bedrock for effective interventions. Common intervention approaches predominantly hinge on ergonomics education, encompassing expert presentations, lectures, and video modules. Simulation-based ergonomics training courses showcase potential benefits in mitigating work-related musculoskeletal injuries. Furthermore, real-time biofeedback presents a promising avenue for intervention, as does postural alignment to circumvent non-neutral postures that exacerbate musculoskeletal disorders. Emerging technologies like augmented reality glasses (ARG) and exoskeleton devices are indicative of the multifaceted approach required to address ergonomic challenges. In video-assisted surgery, the use of ARGs instead of conventional monitor devices can significantly reduce the surgeon’s workload and physical demands, especially for the upper body (66). Moreover, interventions like intraoperative targeted stretching micro breaks (TSMBs), which consisting of five standardized exercises for the neck, shoulders, upper back, lower back, wrists, hands, knees and ankles involving flexion, extension and lateral rotation (77), highlighting their potential in reducing surgeon discomfort while enhancing performance and focus. The current demand for ergonomic interventions signals the need for adequate supply, necessitating innovative integration of human factors engineering and human-machine interaction technologies for more effective and intelligent feedback mechanisms to optimize surgical safety (78).

## 4 Discussion

This review aimed to comprehensively synthesize the literature concerning the objective assessment and quantification of ergonomics in endoscopic surgery, with the goal of constructing a robust intraoperative assessment framework and extrapolating applicable evaluation techniques and settings. By categorizing and elucidating influencing factors, we also evaluated the evidence linking these factors to elevated ergonomics in endoscopic surgery. This pursuit aligns with the domain of human factors engineering, wherein research endeavors strive to understand, evaluate, and optimize human-environment interactions to enhance system performance without compromising human well-being. Modern minimally invasive techniques, like endoscopy, afford patients the advantages of convenience, efficiency, and reduced invasiveness. However, for endoscopic surgeons, particularly given the prevalence of work-related musculoskeletal disorders (WMSDs), the associated physical health risks are paramount. Notably, these risks extend beyond the individual surgeon, as discomfort resulting from human-machine system variables can reverberate into the surgical performance and patient safety (79). Thus, discerning the impact of evolving human-system dynamics on ergonomics and exhaustively quantifying workload and ergonomic factors within the surgical specialty become paramount.

This review takes a comprehensive approach by encompassing various endoscopic specialties, addressing a significant gap in the literature. This expansion beyond the prevailing focus on endoscopic procedures allows for a deeper understanding of ergonomic challenges in diverse surgical domains, including laryngoscopy, nasal endoscopy, thoracoscopy, arthroscopy, and gynecologic endoscopy. In contrast to past studies, our review stands out in its primary focus on “objective” ergonomics assessment, a distinctive feature that has been absent in many previous discussions. Thirdly, we categorize physical and psychological loads disparities into three overarching factors, providing a structured framework for understanding and addressing these issues, thus identifying specific areas for intervention. Fourthly, we underscore the integration of multiple assessment methods, such as motion analysis, surface electromyography, and heart rate variability, resulting in a multi-faceted analysis of ergonomic risks that enriches the evaluation of ergonomic well-being. Finally, our discussion of intervention strategies, including ergonomics education, simulation-based training, real-time biofeedback, and emerging technologies like augmented reality glasses and exoskeleton devices, not only identifies potential solutions but also acknowledges the dynamic nature of the field. In sum, our article offers a holistic understanding of ergonomic challenges, provides a structured framework, and offers practical insights for addressing these issues, making a significant contribution to the field of endoscopic surgery ergonomics.

The prevailing landscape of objective ergonomic evaluations predominantly centers around general surgical applications associated with laparoscopic procedures. Nevertheless, a notable paucity persists in the comprehensive and unbiased quantitative exploration of other endoscopic specialties, including laryngoscopy, nasal endoscopy, thoracoscopy, arthroscopy, and gynecologic endoscopy. These domains warrant further investigation to foster a holistic understanding of ergonomic challenges. Challenges persist in task design, data collection, and analysis complexity, underscoring the need for rigorous investigations that transcend opportunistic research designs characterized by limited sample sizes and short observation periods. Enhancing generalizability and reliability mandates robust study designs with larger cohorts and extended observation windows, across a wider spectrum of endoscopic procedures to capture the intricacies of diverse surgical specialties.

Ergonomics disparities are primarily shaped by three overarching factors: the work task, characteristics related variables, and environmental influences. Existing literature suggests the superiority of laparoscopic surgery over traditional open procedures in terms of both surgical performance and surgeon well-being. Furthermore, robotic-assisted endoscopic surgery emerges as a means to alleviate physical demands on surgeons. The selection of surgery type should align with patient needs, financial considerations, and potential muscle injuries. Delving into surgeon-related factors, optimizing for risk factors such as surgical posture becomes vital, with implications for improved performance distribution and strategic allowances. Gender disparities in ergonomic risks, as evidenced by higher physical loads endured by female surgeons during endoscopic surgeries, underscore the need for tailored interventions like additional breaks and performance considerations. In considering patient-related factors, meticulous preoperative planning must factor in physiological attributes like position and BMI, striving to minimize additional physical stress on the surgeon. Environmental factors, amenable to manipulation, offer opportunities to optimize surgical team arrangement, bed height, instrument positioning, and related parameters through anthropometric principles (Table 3).

**Table 3:** Electromyography-based ergonomic risk assessment.

Study ID	EMG sensors	Muscle selection
Liang (13)	Trigno Wireless EMG	Left and right biceps, triceps, deltoid, and trapezius muscle groups
Ramakrishnan (16)	Noninvasive surface	Medial deltoid; upper trapezius; erector spinae; and biceps femoris
Dalager (18)	Silver/silver, chloride	Right extensor carpi ulnaris, right extensor carpi radialis, right flexor Digitorum superficialis and left upper trapezius
Armijo (19)	Trigno Wireless EMG	Upper trapezius, anterior deltoid, flexor carpi radialis, and extensor digitorum
Rodrigues (21)	Delsys Trigno EMG	Upper trapezius, anterior deltoid, flexor carpi radialis, and extensor digitorum
Zihni (22)	Great Lakes Neurotechnologies	Bilateral biceps, triceps, deltoids and trapezius muscles
Thurston (23)	Delsys Trigno EMG	Triceps, biceps, neck, shoulder, and lower back
Dalager (25)	Silver/silver, chloride	Trapezius muscles, the upper neck muscles, and the erector spinae muscles, upper neck extensor muscles
Shiang (27)	Trigno Wireless EMG	Flexor carpir adialis, palmaris longus, and flexor carpi ulnaris extensor carpi ulnaris and exten-sor digitorum
Armijo (28)	Delsys Trigno EMG	Upper trapezius, anterior deltoid, flexor carpi radialis, and extensor digitorum
Dalsgaard (29)	MQ16, Marq-Medical	Extensor carpiradialis/extensor digitorum, and flexor carpi radialis muscles, neck extensor muscles, the upper trapezius and erector spinae muscle
Zárate (30)	sEMG electrodes	Biceps, triceps, deltoid, and trapezius muscles
Szeto (31)	The Noraxon MyoSystem	The bilateral cervical erector spinae, upper trapezii and anterior deltoid muscles
Zihni (32)	8-channel Bioradio	Bilateral biceps, triceps, deltoid, and trapezius muscles
Monfared (33)	Model, DataLITE, Biometrics	Trapezius and deltoid muscles
Hubert (34)	Surface electrodes	Flexor digitorum and extensor digitorum trapezius erector spinae
Shergill (35)	FingerTPS Wireless Handing Sensing System/bipolar pre-amplified self-adhesive silver/silver chloride snap electrodes	Extensor carpi radialis and flexor digitorum super ficialis muscles
Riggle (39)	Biometrics SX230 and SG65	The flexor digitorum super ficialis and extensor digitorum communis
Lee (36)	DelsysTM EMG	Biceps, triceps, deltoid, trapezius, flexor carpi ulnaris, extensor digitorum, thenar compartment, and erector spinae
Dai (42)	BTS FREEEMG 300 wireless EMG	Flexor carpi ulnaris, biceps, deltoid and trapezius
Wang (43)	Trigno Wireless EMG	Bilateral biceps, triceps, deltoid, and trapezius muscles
Athanasiadis (46)	model DataLITE	The deltoid and trapeziusmuscles
Nieboer (49)	Porti 32, TMS International	The trapezius and deltoid muscles and brachioradial and abductor pollicis brevis muscles
Moss (51)	Wireless miniature low cost EMG sensor using gold plated dry electrodes	Shoulder muscles
Pérez-Duarte (53)	Biopac Systems	The right forearm flexors and extensors muscles
Baird (56)	Delsys Trigno EMG	Arm, shoulders, back, and legs
Asadi (57)	model DataLITE	The deltoid and trapezius muscles
Steinhilber (60)	THUMEDI, Thum-Jahnsbach	Musculus trapezius pars descendens, musculus deltoideus pars acromialis, musculus biceps brachii (BIC), musculus extensor digitorum, and musculus flexor carpi radialis
Yoon (61)	TeleMyo 2,400 T DTS	The splenius capitis, upper trapezius, middle deltoid, flexor carpi radialis, extensor carpi radialis, lumbar erector spinae, rectus femoralis and tibialis anterior
Zihni (80)	8-channel Bioradio	Bicep, tricep, deltoid and trapezius muscles
Shergill (63)	TeleMyo 2,400 T telemetric EMG system	Extensor carpi radialis, the flexor digitorum superficialis, and the left abductor pollicis longus
Butler (65)	Jamar hydraulic hand grip dynamometer	Dominant hand
Lim (66)	LXM 3208-RF EMG	Sternocleidomastoid, trapezius pars descendens, brachioradialis, erector spinae longissimus
Zhang (67)	Delsys Trigno EMG	Trapezius, bicipital, brachioradialis and flexor carpi ulnaris muscles

The assessment phase mandates comprehensive evaluation encompassing both physiological and psychological burdens on the endoscopic surgeon. Commonly employed methods for objective ergonomic quantification include motion analysis and electromyographic signals. While traditional photogrametry is surpassed by motion analysis, advanced human skeleton prediction techniques rooted in computer visiozn or inertial sensing find broad application. Electromyography (EMG) signals enable the assessment of ergonomic compliance, while quantifying the mental load during endoscopy, vital given its association with WMSDs, relies on indicators such as EEG, ECG, EDA, and eye movement. Harmonizing physical and mental load assessment, coupled with in-depth comprehension of their intricate interplay, holds the potential to enhance overall ergonomic well-being among endoscopic surgeons. It’s worth noting that in the context of assessing the psychological burden on endoscopists, our review identifies a significant gap: the predominant reliance on subjective questionnaires to measure mental load. This is despite acknowledging that such approaches may not capture the full spectrum of cognitive demands. Notably, only one study within our reviewed literature incorporated an objective indicator of psychological load—HRV (37). This highlights a critical deficiency in current research and underscores the potential for future investigations to employ objective methods such as HRV. Advancing this direction could yield more accurate assessments of the mental burden on endoscopists, informing targeted strategies to alleviate this burden and enhance overall patient care.

In our systematic review of ergonomic assessment methods in endoscopic surgery, we identified distinct strengths and limitations for each approach. Motion analysis stands out for its precision in tracking movements but is limited by its need for specialized environments and equipment (83, 84), potentially reducing its practicality in real surgical settings. Electromyography offers direct muscle exertion insights, yet is hindered by susceptibility to signal interference and the challenges of electrode placement in sterile conditions (85). Heart rate variability, while a useful non-invasive mental load indicator (86), lacks specificity to ergonomic stress due to its sensitivity to various factors (87). Similarly, salivary cortisol provides stress biomarkers but is affected by individual variability and diurnal patterns, limiting its immediate relevance to ergonomic assessment. Eye tracking technology, though valuable for measuring attentional focus, does not directly assess ergonomic load and requires careful calibration. These findings underscore the need for a multifaceted and practical approach in ergonomic assessment, integrating both objective measurements and subjective feedback to comprehensively address the ergonomic challenges in surgical environments. Table 4 summarizes the advantages, disadvantages, and limitations of these assessment tool.

**Table 4:** Comparative summary of assessment methods for ergonomics in endoscopic surgery.

Assessment method	Strengths	Weaknesses	Limitations
Motion analysis	Precise tracking of surgeon movements	Resource-heavy and technically demanding	May not fully capture dynamic muscle coordination
Electromyography (EMG)	Direct assessment of muscle activity	Susceptible to signal interference and cross-talk	Electrode placement and external factors can affect readings
Heart rate variability (HRV)	Non-invasive with good stress-response indication	Indirect measure of ergonomic load; sensitive to many variables	Interpretation complexity; confounded by non-ergonomic factors
Salivary cortisol	Stress biomarker that is easy to collect	Individual variability; influenced by time of day	Collection timing critical; not specific to ergonomics
Eye tracking	Objective focus and attention measurement	Does not measure ergonomic load directly	Calibration required; can be invasive for some users

Composite assessment methodologies integrating parameters like motion analysis, surface EMG, and heart rate variability have emerged as vital tools for ergonomic risk assessment. This multi-faceted analysis probes intraoperative ergonomics from diverse dimensions, enriching assessment comprehensiveness. While real-life OR surgery provides high reliability assessments, the associated surgical risks, including unpredictable intraoperative data collection and potential instrument damage, limit its feasibility. Similarly, while cadaveric surgery offers a high-fidelity assessment environment, practical constraints like expense and scarcity hamper its widespread application. Ergonomic evaluation within surgical simulator environments emerges as a viable alternative, albeit with a current focus on novice training rather than comprehensive ergonomic exploration. This gap warrants addressing for optimal integration into surgical practice.

## 5 Conclusion

The review identified three primary categories influencing ergonomic load in endoscopic surgery: environmental factors, characteristics-related factors and factors related to work task and proposed an overall research framework. The studies revealed that ergonomic challenges are prevalent in real and simulated surgical environments, with a significant impact on workloads. Our analysis underscores the multifaceted nature of ergonomic risks in endoscopic procedures. It highlights the importance of considering the entire surgical ecosystem, including the surgical team, equipment, patient characteristics, and the specific surgical procedure, to effectively address these challenges. The findings from this comprehensive review serve as a pivotal guide for future ergonomic assessments and interventions aimed at enhancing surgeon well-being and patient safety in endoscopic surgery.

## Data availability statement

The original contributions presented in the study are included in the article/supplementary material, further inquiries can be directed to the corresponding authors.

## Author contributions

SL: Conceptualization, Data curation, Writing – original draft. Y-YL: Data curation, Visualization, Writing – original draft. DL: Data curation, Writing – original draft. F-YW: Writing – review & editing. L-JF: Writing – review & editing. L-xZ: Writing – review & editing.

## References

[1] Sakai P Faintuch J. Evolving endoscopic surgery. J Gastroenterol Hepatol. (2014) 29:1132–8. doi: 10.1111/jgh.12577 24628672

[2] Park A Lee G Seagull FJ Meenaghan N Dexter D. Patients benefit while surgeons suffer: an impending epidemic. J Am Coll Surg. (2010) 210:306–13. doi: 10.1016/j.jamcollsurg.2009.10.017, PMID: 20193893

[3] Catanzarite T Tan-Kim J Whitcomb EL Menefee S. Ergonomics in surgery: a review. Female Pelvic Med Reconstr Surg. (2018) 24:1–12. doi: 10.1097/SPV.0000000000000456, PMID: 28914699

[4] Alleblas CCJ de Man AM van den Haak L Vierhout ME Jansen FW Nieboer TE. Prevalence of musculoskeletal disorders among surgeons performing minimally invasive surgery: a systematic review. Ann Surg. (2017) 266:905–20. doi: 10.1097/SLA.0000000000002223 28306646

[5] Szeto G Ho P Ting A Poon J Cheng S Tsang R. Work-related musculoskeletal symptoms in surgeons. J Occup Rehabil. (2009) 19:175–84. doi: 10.1007/s10926-009-9176-1 19381790

[6] Davis WT Fletcher SA Guillamondegui OD. Musculoskeletal occupational injury among surgeons: effects for patients, providers, and institutions. J Surg Res. (2014) 189:207–212.e6. doi: 10.1016/j.jss.2014.03.013, PMID: 24721601

[7] Lowndes BR Hallbeck MS. Overview of human factors and ergonomics in the OR, with an emphasis on minimally invasive surgeries. Hum Factors Ergon Manuf Serv Ind. (2014) 24:308–17. doi: 10.1002/hfm.20383

[8] International Ergonomics Association (IEA) Council . Definition and domains of ergonomics. Available at: http://www.iea.cc/whats/index.html (Accessed January 22, 2016).

[9] Seagull FJ . Disparities between industrial and surgical ergonomics. Work. (2012) 41:4669–72. doi: 10.3233/WOR-2012-0107-4669, PMID: 22317439

[10] Lipowska AM Shergill AK. Ergonomics of endoscopy. Gastrointest Endosc Clin N Am. (2021) 31:655–69. doi: 10.1016/j.giec.2021.05.003 34538406

[11] Kim SE Junggi Hong PDATC. Ergonomic interventions as a treatment and preventative tool for work-related musculoskeletal disorders. Int J Caring Sci. (2013) 6:339.

[12] David GC . Ergonomic methods for assessing exposure to risk factors for work-related musculoskeletal disorders. Occup Med. (2005) 55:190–9. doi: 10.1093/occmed/kqi082 15857898

[13] Liang Z Gerull WD Wang R Zihni A Ray S Awad M. Effect of patient body mass index on laparoscopic surgical ergonomics. Obes Surg. (2019) 29:1709–13. doi: 10.1007/s11695-019-03748-0, PMID: 30712169

[14] Sers R Forrester S Zecca M Ward S Moss E. The ergonomic impact of patient body mass index on surgeon posture during simulated laparoscopy. Appl Ergon. (2021) 97:103501. doi: 10.1016/j.apergo.2021.103501 34167015

[15] Arrighi-Allisan AE Garvey KL Wong A Filip P Shah J Spock T . Ergonomic analysis of functional endoscopic sinus surgery using novel inertial sensors. Laryngoscope. (2022) 132:1153–9. doi: 10.1002/lary.29796, PMID: 34355793

[16] Ramakrishnan VR Milam BM. Ergonomic analysis of the surgical position in functional endoscopic sinus surgery. Int Forum Allergy Rhinol. (2017) 7:570–5. doi: 10.1002/alr.21911, PMID: 28296272

[17] Lobo D Anuarbe P López-Higuera JM Viera J Castillo N Megía R. Estimation of surgeons’ ergonomic dynamics with a structured light system during endoscopic surgery. Int Forum Allergy Rhinol. (2019) 9:857–64. doi: 10.1002/alr.22353, PMID: 31090195

[18] Dalager T Jensen PT Eriksen JR Jakobsen HL Mogensen O Søgaard K. Surgeons' posture and muscle strain during laparoscopic and robotic surgery. Br J Surg. (2020) 107:756–66. doi: 10.1002/bjs.11394, PMID: 31922258

[19] Armijo PR Flores L Pokala B Huang CK Siu KC Oleynikov D. Gender equity in ergonomics: does muscle effort in laparoscopic surgery differ between men and women? Surg Endosc. (2022) 36:396–401. doi: 10.1007/s00464-021-08295-3, PMID: 33492502

[20] Kratzke IM Zhou G Mosaly P Farrell TM Crowner J Yu D. Evaluating the ergonomics of surgical residents during laparoscopic simulation: a novel computerized approach. Am Surg. (2022) 89:1622–8. doi: 10.1177/00031348211047505 35045763

[21] Rodrigues Armijo P Huang CK Carlson T Oleynikov D Siu KC. Ergonomics analysis for subjective and objective fatigue between laparoscopic and robotic surgical skills practice among surgeons. Surg Innov. (2020) 27:81–7. doi: 10.1177/1553350619887861, PMID: 31771411

[22] Zihni AM Cavallo JA Ray S Ohu I Cho S Awad MM. Ergonomic analysis of primary and assistant surgical roles. J Surg Res. (2016) 203:301–5. doi: 10.1016/j.jss.2016.03.058, PMID: 27363636

[23] Thurston T Dolan JP Husein F Stroud A Funk K Borzy C . Assessment of muscle activity and fatigue during laparoscopic surgery. Surg Endosc. (2022) 36:1–7. doi: 10.1007/s00464-021-08937-6 35034217

[24] Lowndes BR Abdelrahman AM Thiels CA Mohamed AO McConico AL Bingener J . Surgical team workload comparison for 4-port and single-port laparoscopic cholecystectomy procedures. Appl Ergon. (2019) 78:277–85. doi: 10.1016/j.apergo.2018.06.005, PMID: 29960648

[25] Dalager T Jensen PT Winther TS Savarimuthu TR Markauskas A Mogensen O . Surgeons’ muscle load during robotic-assisted laparoscopy performed with a regular office chair and the preferred of two ergonomic chairs: a pilot study. Appl Ergon. (2019) 78:286–92. doi: 10.1016/j.apergo.2018.03.016, PMID: 29650223

[26] Lohre R Regan W Goel DP. Surgeon ergonomics during arthroscopic shoulder surgery. J Orthop Exp Innov. (2020) 1:13307. doi: 10.60118/001c.13307

[27] Shiang A Wang JS Kushner B Panahi AK Awad MM. Smaller hands and less experience are associated with greater ergonomic strain during endoscopic procedures. Surg Endosc. (2022) 36:5104–9. doi: 10.1007/s00464-021-08876-2, PMID: 34845543

[28] Armijo PR Huang CK High R Leon M Siu KC Oleynikov D. Ergonomics of minimally invasive surgery: an analysis of muscle effort and fatigue in the operating room between laparoscopic and robotic surgery. Surg Endosc. (2019) 33:2323–31. doi: 10.1007/s00464-018-6515-3, PMID: 30341653

[29] Dalsgaard T Jensen MD Hartwell D Mosgaard BJ Jørgensen A Jensen BR. Robotic surgery is less physically demanding than laparoscopic surgery: paired cross sectional study. Ann Surg. (2020) 271:106–13. doi: 10.1097/SLA.0000000000002845, PMID: 29923873

[30] Zárate Rodriguez JG Zihni AM Ohu I Cavallo JA Ray S Cho S . Ergonomic analysis of laparoscopic and robotic surgical task performance at various experience levels. Surg Endosc. (2019) 33:1938–43. doi: 10.1007/s00464-018-6478-4, PMID: 30350099

[31] Szeto GPY Poon JTC Law WL. A comparison of surgeon’s postural muscle activity during robotic-assisted and laparoscopic rectal surgery. J Robot Surg. (2013) 7:305–8. doi: 10.1007/s11701-012-0374-z, PMID: 27000928

[32] Zihni AM Ohu I Cavallo JA Cho S Awad MM. Ergonomic analysis of robot-assisted and traditional laparoscopic procedures. Surg Endosc. (2014) 28:3379–84. doi: 10.1007/s00464-014-3604-9, PMID: 24928233

[33] Monfared S Athanasiadis DI Umana L Hernandez E Asadi H Colgate CL . A comparison of laparoscopic and robotic ergonomic risk. Surg Endosc. (2022) 36:1–6. doi: 10.1007/s00464-022-09105-0 35182219

[34] Hubert N Gilles M Desbrosses K Meyer JP Felblinger J Hubert J. Ergonomic assessment of the surgeon's physical workload during standard and robotic assisted laparoscopic procedures. Int J Med Robot. (2013) 9:142–7. doi: 10.1002/rcs.1489, PMID: 23529792

[35] Shergill AK Rempel D Barr A Lee D Pereira A Hsieh CM . Biomechanical risk factors associated with distal upper extremity musculoskeletal disorders in endoscopists performing colonoscopy. Gastrointest Endosc. (2021) 93:704–711.e3. doi: 10.1016/j.gie.2020.11.001 33160978

[36] Lee D L Rempel D Barr A B Shergill A Ergonomics of colonoscopy: wrist postures of gastroenterologists performing routine colonoscopy. Proceedings of the Human Factors and Ergonomics Society Annual Meeting. Los Angeles, CA: SAGE Publications, (2010), 54: 1205–1209.

[37] Abdelrahman AM Bingener J Yu D Lowndes BR Mohamed A McConico AL . Impact of single-incision laparoscopic cholecystectomy (SILC) versus conventional laparoscopic cholecystectomy (CLC) procedures on surgeon stress and workload: a randomized controlled trial. Surg Endosc. (2016) 30:1205–11. doi: 10.1007/s00464-015-4332-5, PMID: 26194249 PMC4721929

[38] Hallbeck MS Lowndes BR McCrory B Morrow MM Kaufman KR LaGrange CA. Kinematic and ergonomic assessment of laparoendoscopic single-site surgical instruments during simulator training tasks. Appl Ergon. (2017) 62:118–30. doi: 10.1016/j.apergo.2017.02.003 28411722

[39] Riggle JD Miller EE McCrory B Meitl A Lim E Hallbeck MS . Ergonomic comparison of laparoscopic hand instruments in a single site surgery simulator with novices. Minim Invasive Ther Allied Technol. (2015) 24:68–76. doi: 10.3109/13645706.2014.946426, PMID: 25142199

[40] Lee GI Lee MR Clanton T Sutton E Park AE Marohn MR. Comparative assessment of physical and cognitive ergonomics associated with robotic and traditional laparoscopic surgeries. Surg Endosc. (2014) 28:456–65. doi: 10.1007/s00464-013-3213-z, PMID: 24196542

[41] Yang L Wang T Weidner TK Madura JA II Morrow MM Hallbeck MS. Intraoperative musculoskeletal discomfort and risk for surgeons during open and laparoscopic surgery. Surg Endosc. (2021) 35:6335–43. doi: 10.1007/s00464-020-08085-3, PMID: 33083930

[42] Dai X Fan S Hao H Yang K Shen C Xiong G . Comparison of KD-SR-01 robotic partial nephrectomy and 3D-laparoscopic partial nephrectomy from an operative and ergonomic perspective: a prospective randomized controlled study in porcine models. Int J Med Robot. (2021) 17:e2187. doi: 10.1002/rcs.2187, PMID: 33068498

[43] Wang R Liang Z Zihni AM Ray S Awad MM. Which causes more ergonomic stress: laparoscopic or open surgery? Surg Endosc. (2017) 31:3286–90. doi: 10.1007/s00464-016-5360-5 27924389

[44] Pazouki A Sadati L Zarei F Golchini E Fruzesh R Bakhtiary J. Ergonomic challenges encountered by laparoscopic surgeons, surgical first assistants, and operating room nurses involved in minimally invasive surgeries by using RULA method. J Minim Invasive Surg Sci. (2017) 6:344–6. doi: 10.5812/minsurgery.60053

[45] Hignett S Moss E L Gyi D Calkins L Jones LL. Save our surgeons: an ergonomics evaluation of laparoscopic hysterectomy. Loughborough University. Conference contribution (2017). Available at: https://hdl.handle.net/2134/23674

[46] Athanasiadis DI Monfared S Asadi H Colgate CL Yu D Stefanidis D. An analysis of the ergonomic risk of surgical trainees and experienced surgeons during laparoscopic procedures. Surgery. (2021) 169:496–501. doi: 10.1016/j.surg.2020.10.027, PMID: 33246648

[47] Bartnicka J Zietkiewicz AA Kowalski GJ. An ergonomics study on wrist posture when using laparoscopic tools in four techniques in minimally invasive surgery. Int J Occup Saf Ergon. (2018) 24:438–49. doi: 10.1080/10803548.2018.1452666, PMID: 29553920

[48] McCrory B Lowndes BR Wirth LM de Laveaga AE LaGrange CA Hallbeck MS. Ergonomic evaluation of laparoendoscopic single-site surgery ports in a validated laparoscopic training model. Work. (2012) 41:1884–90. doi: 10.3233/WOR-2012-0402-1884, PMID: 22316990

[49] Nieboer TE Massa M Weinans MJN Vierhout ME Kluivers KB Stegeman DF. Does training of the nondominant upper extremity reduce the surgeon’s muscular strain during laparoscopy? Results from a randomized controlled trial. Surg Innov. (2013) 20:292–8. doi: 10.1177/1553350612456099, PMID: 22918936

[50] Yu D Lowndes B Morrow M Kaufman K Bingener J Hallbeck S. Impact of novel shift handle laparoscopic tool on wrist ergonomics and task performance. Surg Endosc. (2016) 30:3480–90. doi: 10.1007/s00464-015-4634-7, PMID: 26541720 PMC4860168

[51] Moss EL Sarhanis P Ind T Smith M Davies Q Zecca M. Impact of obesity on surgeon ergonomics in robotic and straight-stick laparoscopic surgery. J Minim Invasive Gynecol. (2020) 27:1063–9. doi: 10.1016/j.jmig.2019.07.009, PMID: 31326633

[52] Sánchez-Margallo FM Pérez-Duarte FJ Sánchez-Margallo JA Lucas-Hernández M Matos-Azevedo AM Díaz-Güemes I. Application of a motion capture data glove for hand and wrist ergonomic analysis during laparoscopy. Minim Invasive Ther Allied Technol. (2014) 23:350–6. doi: 10.3109/13645706.2014.925928, PMID: 24910159

[53] Pérez-Duarte FJ Sánchez-Margallo FM Martín-Portugués IDG Sánchez-Hurtado MA Lucas-Hernández M Sánchez-Margallo JA . Ergonomic analysis of muscle activity in the forearm and back muscles during laparoscopic surgery: influence of previous experience and performed task. Surg Laparosc Endosc Percutan Tech. (2013) 23:203–7. doi: 10.1097/SLE.0b013e3182827f30, PMID: 23579519

[54] Yang L Money SR Morrow MM Lowndes BR Weidner TK Fortune E . Impact of procedure type, case duration, and adjunctive equipment on surgeon intraoperative musculoskeletal discomfort. J Am Coll Surg. (2020) 230:554–60. doi: 10.1016/j.jamcollsurg.2019.12.035, PMID: 32220445

[55] Khan R Scaffidi MA Satchwell J Gimpaya N Lee W Genis S . Impact of a simulation-based ergonomics training curriculum on work-related musculoskeletal injury risk in colonoscopy. Gastrointest Endosc. (2020) 92:1070–1080.e3. doi: 10.1016/j.gie.2020.03.3754 32205194

[56] Baird BJ Tynan MA Tracy LF Heaton JT Burns JA. Surgeon positioning during awake laryngeal surgery: an ergonomic analysis. Laryngoscope. (2021) 131:2752–8. doi: 10.1002/lary.29717, PMID: 34296439

[57] Asadi H Monfared S Athanasiadis DI Stefanidis D Yu D. Continuous, integrated sensors for predicting fatigue during non-repetitive work: demonstration of technique in the operating room. Ergonomics. (2021) 64:1160–73. doi: 10.1080/00140139.2021.1909753, PMID: 33974511

[58] Yurteri-Kaplan LA Zhu X Iglesia CB Gutman RE Sokol AI Paquet V . Differences in postural loading between primary and assistant surgeons during vaginal surgery. Int J Ind Ergon. (2018) 65:60–7. doi: 10.1016/j.ergon.2018.01.003

[59] Ordóñez-Ríos M Jara-Díaz O Salamea J C Robles-Bykbaev V. Ergonomic assessment and analysis of postural load of surgeons performing laparoscopic surgeries in Cuenca, Ecuador. International conference on applied human factors and ergonomics. Springer, Cham, (2017): 427–437.

[60] Steinhilber B Reiff F Seibt R Rieger MA Martus P Kraemer B . Ergonomic benefits from a laparoscopic instrument with rotatable handle piece depend on the area of the operating field and working height. Hum Factors. (2017) 59:1048–65. doi: 10.1177/0018720817712597 28628750

[61] Yoon SH Jung MC Park SY. Evaluation of surgeon’s muscle fatigue during thoracoscopic pulmonary lobectomy using interoperative surface electromyography. J Thorac Dis. (2016) 8:1162–9. doi: 10.21037/jtd.2016.04.16, PMID: 27293833 PMC4886009

[62] Hardy NP Mannion J Johnson R Greene G Hehir DJ. In vivo assessment of cervical movement in surgeons—results from open and laparoscopic procedures. Ir J Med Sci. (2021) 190:269–73. doi: 10.1007/s11845-020-02255-x 32500446

[63] Shergill AK Asundi KR Barr A Shah JN Ryan JC McQuaid KR . Pinch force and forearm-muscle load during routine colonoscopy: a pilot study. Gastrointest Endosc. (2009) 69:142–6. doi: 10.1016/j.gie.2008.09.030, PMID: 19111694

[64] Pace-Bedetti HM Dolz JF Martínez-de-Juan JL Conejero A. The effect of postural freedom to increase the neutral positions during laparoscopic surgery. Int J Interact Des Manuf. (2019) 13:627–31. doi: 10.1007/s12008-018-00527-6

[65] Butler KA Kapetanakis VE Smith BE Sanjak M Verheijde JL Chang YHH . Surgeon fatigue and postural stability: is robotic better than laparoscopic surgery? J Laparoendosc Adv Surg Tech. (2013) 23:343–6. doi: 10.1089/lap.2012.0531, PMID: 23410117

[66] Lim AK Ryu J Yoon HM Yang HC Kim SK. Ergonomic effects of medical augmented reality glasses in video-assisted surgery. Surg Endosc. (2022) 36:988–98. doi: 10.1007/s00464-021-08363-8, PMID: 33638103

[67] Zhang JY Liu SL Feng QM Gao JQ Zhang Q. Correlative evaluation of mental and physical workload of laparoscopic surgeons based on surface electromyography and eye-tracking signals. Sci Rep. (2017) 7:1–7. doi: 10.1038/s41598-017-11584-4 28894216 PMC5594030

[68] Hart SG Staveland LE. Development of NASA-TLX (task load index): results of empirical and theoretical research In: Hancock PA Meshkati N, editors. Human mental workload. Amsterdam: North Holland Press (1988)

[69] Craven R Franasiak J Mosaly P Gehrig PA. Ergonomic deficits in robotic gynecologic oncology surgery: a need for intervention. J Minim Invasive Gynecol. (2013) 20:648–55. doi: 10.1016/j.jmig.2013.04.008, PMID: 23747116

[70] van Veelen MA Kazemier G Koopman J Goossens RHM Meijer DW. Assessment of the ergonomically optimal operating surface height for laparoscopic surgery. J Laparoendosc Adv Surg Tech. (2002) 12:47–52. doi: 10.1089/109264202753486920, PMID: 11908485

[71] Rodman C Kelly N Niermeyer W Banks L Onwuka A Mason E . Quantitative assessment of surgical ergonomics in otolaryngology. Otolaryngol Head Neck Surg. (2020) 163:1186–93. doi: 10.1177/0194599820932851, PMID: 32600215

[72] McAtamney L Corlett EN. RULA: a survey method for the investigation of work-related upper limb disorders. Appl Ergon. (1993) 24:91–9. doi: 10.1016/0003-6870(93)90080-S, PMID: 15676903

[73] Hignett S McAtamney L. Rapid entire body assessment (REBA). Appl Ergon. (2000) 31:201–5. doi: 10.1016/S0003-6870(99)00039-3 10711982

[74] Kee D Karwowski W. LUBA: an assessment technique for postural loading on the upper body based on joint motion discomfort and maximum holding time. Appl Ergon. (2001) 32:357–66. doi: 10.1016/S0003-6870(01)00006-0, PMID: 11461037

[75] Beatty J . Task-evoked pupillary responses, processing load, and the structure of processing resources. Psychol Bull. (1982) 91:276–92. doi: 10.1037/0033-2909.91.2.276, PMID: 7071262

[76] Bjerrum F Thomsen ASS Nayahangan LJ Konge L. Surgical simulation: current practices and future perspectives for technical skills training. Med Teach. (2018) 40:668–75. doi: 10.1080/0142159X.2018.1472754, PMID: 29911477

[77] Park AE Zahiri HR Hallbeck MS Augenstein V Sutton E Yu D . Intraoperative “micro breaks” with targeted stretching enhance surgeon physical function and mental focus. Ann Surg. (2017) 265:340–6. doi: 10.1097/SLA.0000000000001665, PMID: 28059962

[78] Epstein S Sparer EH Tran BN Ruan QZ Dennerlein JT Singhal D . Prevalence of work-related musculoskeletal disorders among surgeons and interventionalists: a systematic review and meta-analysis. JAMA Surg. (2018) 153:e174947–7. doi: 10.1001/jamasurg.2017.4947, PMID: 29282463 PMC5838584

[79] Albanesi B Piredda M Bravi M Bressi F Gualandi R Marchetti A . Interventions to prevent and reduce work-related musculoskeletal injuries and pain among healthcare professionals. A comprehensive systematic review of the literature. J Saf Res. (2022) 82:124–43. doi: 10.1016/j.jsr.2022.05.004, PMID: 36031239

[80] Zihni AM Ohu I Cavallo JA Ousley J Cho S Awad MM. FLS tasks can be used as an ergonomic discriminator between laparoscopic and robotic surgery. Surg Endosc. (2014) 28:2459–65. doi: 10.1007/s00464-014-3497-7 24619332

[81] Wade L Needham L McGuigan P Bilzon J. Applications and limitations of current markerless motion capture methods for clinical gait biomechanics. PeerJ. (2022) 10:e12995. doi: 10.7717/peerj.12995, PMID: 35237469 PMC8884063

[82] Muro-De-La-Herran A Garcia-Zapirain B Mendez-Zorrilla A. Gait analysis methods: an overview of wearable and non-wearable systems, highlighting clinical applications. Sensors. (2014) 14:3362–94. doi: 10.3390/s140203362, PMID: 24556672 PMC3958266

[83] al-Ayyad M Owida HA de Fazio R al-Naami B Visconti P. Electromyography monitoring Systems in Rehabilitation: a review of clinical applications, wearable devices and signal acquisition methodologies. Electronics. (2023) 12:1520. doi: 10.3390/electronics12071520

[84] van Amelsvoort LGPM Schouten EG Maan AC Swenne CA Kok FJ. Occupational determinants of heart rate variability. Int Arch Occup Environ Health. (2000) 73:255–62. doi: 10.1007/s004200050425 10877031

[85] Tiwari R Kumar R Malik S Raj T Kumar P. Analysis of heart rate variability and implication of different factors on heart rate variability. Curr Cardiol Rev. (2021) 17:74–83. doi: 10.2174/1573403X16999201231203854 33390146 PMC8950456

[86] Owlia M Kamachi M Dutta T. Reducing lumbar spine flexion using real-time biofeedback during patient handling tasks. Work. (2020) 66:41–51. doi: 10.3233/WOR-203149, PMID: 32417812 PMC7369082

[87] Yu D Dural C Morrow M Yang L Collins JW Hallbeck S . Intraoperative workload in robotic surgery assessed by wearable motion tracking sensors and questionnaires. Surg Endosc. (2017) 31:877–86. doi: 10.1007/s00464-016-5047-y, PMID: 27495330

